# Dietary Yellow Bells (*Tecoma stans*) Flower Enhances Gut Health, Fillet Quality, Hematological Indices, and Whole-Body Composition in Nile Tilapia (*Oreochromis niloticus*)

**DOI:** 10.3390/ani16111702

**Published:** 2026-06-02

**Authors:** Kanokwan Hyukhongkaeo, Nutt Nuntapong, Waraporn Hahor, Karun Thongprajukaew

**Affiliations:** 1 Applied Aquatic Animal Nutrition Laboratory, Division of Health and Applied Sciences, Faculty of Science, Prince of Songkla University, Songkhla 90110, Thailand; 6610210520@psu.ac.th (K.H.); waraporn.hahor@gmail.com (W.H.); 2 Kidchakan Supamattaya Aquatic Animal Health Research Center, Aquatic Science and Innovative Management Division, Faculty of Natural Resources, Prince of Songkla University, Songkhla 90110, Thailand; nutt.n@psu.ac.th

**Keywords:** edible flower, feed supplement, herb, yellow trumpetbush, *T. stans*

## Abstract

Yellow bells (*Tecoma stans*) is a popular ornamental flowering plant used in public spaces. Its flowers are considered a medicinal herb rich in bioactive compounds. This study aimed to investigate the effect of using dried yellow bells flower (YB) as a dietary supplement for Nile tilapia (*Oreochromis niloticus*). Nile tilapia (0.74 ± 0.01 g body weight) were divided into six groups for an eight-week feeding trial, during which their diets were supplemented with 5 different levels by weight of YB: 2, 4, 6, 8, and 10%. The control group received a non-supplemented diet. Parameters related to growth, feed utilization, skin and fillet coloration, gut functionality, fillet quality, blood parameters, and whole-body composition were observed. Survival, feed utilization, and skin and fillet coloration were unaffected by YB supplementation, and growth performance was generally maintained up to 8% dietary inclusion. However, at 4 to 6% YB intestinal cellulase activity and gastrointestinal radical-scavenging activities were significantly increased, while amylase and protease activities, and the amylase/trypsin ratio, were maintained. Fillet quality was improved at 4% YB, with higher myosin and total myofibrillar protein contents but without changes in RNA, total protein, or RNA/protein ratio. At moderate YB inclusion levels, white blood cell counts were lower and packed cell volume and hemoglobin levels were higher, indicating improved physiological status. Whole-body crude protein and ash were increased at higher YB levels, whereas moisture and lipid were unchanged. Overall, dietary inclusion of around 4% YB was associated with favorable improvements in gut functionality, fillet quality, blood parameters, and whole-body composition in Nile tilapia.

## Simple Summary

Yellow bells is a common ornamental plant whose flowers contain natural compounds that may improve animal health. This study tested dried yellow bells flowers as a feed supplement for Nile tilapia, an important farmed fish. Young fish were fed diets containing different levels of flower powder for eight weeks. Moderate inclusion levels did not reduce survival, growth, or feed utilization, but improved digestive function and antioxidant protection in the gut. Fish given these diets also showed better flesh quality and healthier blood values, indicating improved overall condition. Higher dietary levels increased body protein and mineral content, while body fat and moisture were unchanged. Among the tested diets, the diet containing approximately 4% yellow bells flower showed the most favorable overall responses. These findings show that yellow bells flowers may be a low-cost natural feed ingredient to improve fish health and product quality while supporting more sustainable aquaculture.

## 1. Introduction

Yellow bells (*Tecoma stans*; YB) is a drought-tolerant ornamental shrub widely cultivated in tropical and subtropical regions for urban greening and landscape beautification. Under tropical environmental conditions, the plant can flower almost continuously throughout the year, although flowering intensity may increase during warm and rainy seasons [1,2]. Mature plants commonly reach 3–8 m in height and produce abundant yellow flowers in dense terminal clusters, particularly in roadside plantings, parks, and residential landscapes. In Thailand, where the species is widely planted as an ornamental hedge and roadside tree, substantial amounts of flowers can be collected regularly from natural flower abscission and routine pruning activities. Although precise quantitative data on annual flower biomass production remain limited, the prolonged flowering habit and widespread cultivation of *T. stans* suggest that YB flower may represent a sustainable and readily available phytogenic resource for aquaculture applications.

Traditionally, *T. stans* flowers have been used in herbal medicine for the management of diabetes and gastrointestinal disorders [3,4,5]. These traditional uses are effective because YB and other species in the genus Tecoma contain a variety of bioactive compounds, including saponins, flavonoids, alkaloids, phenols, steroids, anthraquinones, tannins, terpenes, hydrocarbons, essential oils, glycosylated flavonoids, and phenolic acids [4,5,6,7]. These bioactive compounds are known to be effective in the treatment of diseases related to the digestive system [5,8]. Studies have also found that the active compounds in the flowers of YB have potential as anti-inflammatory and anti-cancer agents against breast cancer, lung cancer, colon cancer, and rectal cancer [9,10,11] and as anti-fungal and anti-bacterial agents against pathogenic bacteria such as *Aspergillus flavus*, *Candida albicans*, *Aeromonas* spp., *Escherichia coli*, and *Enterococcus faecalis* [4,12]. YB flowers also contain coumarin and anthocyanin, which are important antioxidants [10]. However, the use of YB flower as an additive in aquaculture feed has not been reported.

In the aquaculture industry, the use of antibiotics is no longer acceptable due to the negative long-term impact of residual antibiotics on the health of consumers [13,14]. The World Health Organization promotes the use of herbal plants as feed additives in place of antibiotics [14,15]. In the past, marigolds (*Tagetes erecta*) were used as an alternative raw material for aquaculture feeds because marigolds are inexpensive and bloom almost all year round [16]. They also contain bioactive compounds with anti-bacterial and anti-oxidant properties [17]. It was found that dietary supplementation with marigolds helped control diseases of fish and shrimp, promote their growth, stimulate their appetite, and boost their immunity [17]. In addition, marigold supplementation at a dose of 20 mg/kg helped improve the color intensity of the grass carp (*Cyprinus carpio*) and the banana fish (*Labidochromis caeruleus*) [16,18]. The color intensity of the fish was enhanced because marigold flowers contain a high concentration of lutein, a compound in the carotenoid group [16]. The reported data and past use of marigolds in aquaculture, and the known applications of YB, gave our team the idea to use YB, a flowering plant with similar color and phytochemicals to the marigold, as an alternative feed additive in aquaculture.

Nile tilapia (*Oreochromis niloticus*) is a freshwater fish native to tropical and subtropical Africa, including East, Central, and West regions [19,20]. Widely farmed, Nile tilapia is a fast-growing, disease-resistant fish that adapts to diverse environments, reproduces well, is easy to raise, provides high-quality meat, is inexpensive, and requires low nutrient inputs [21]. Since it is mostly raised in intensive production systems, special care is required in certain aspects of its cultivation. The feed used should offer complete nutritional content as well as economic and environmental sustainability. Feed additives must be used to enhance growth efficiency, feed utilization, and improve health [22]. Since tilapia can efficiently utilize waste or biological by-products, previous additives used in tilapia feed have included flowering plants, such as ground chamomile (*Matricaria chamomilla*), ground lavender (*Lavandula angustifolia*), moringa (*Moringa oleifera*) and ground banana inflorescence (*Musa* sp.) [22,23,24].

This study aimed to evaluate the biological responses of Nile tilapia to different dietary inclusion levels of dried YB flower as a feed additive. The findings are expected to support the utilization of YB flowers as a functional plant-based feed ingredient. In addition, the use of YB flowers may provide an alternative value-added application for this widely available ornamental plant.

## 2. Materials and Methods

### 2.1. Nutritional Composition

#### 2.1.1. Dried YB Flower Preparation

Fresh YB flowers were collected from the campus of Prince of Songkla University, Songkhla Province, during November 2024 to January 2025. The flowers were cleaned in 5 mg/L of potassium permanganate for 10 min and washed twice with tap water. The cleaned petals were dried at 50 °C for 24 h, ground and sieved into a fine powder. The dry powder was placed in transparent polyethylene bags that were then wrapped in aluminum foil to exclude light, and kept at 4 °C in the dark before the analysis of proximate chemical composition, total carotenoid contents, and in vitro digestibility. To enable a comparison of proximate chemical composition and in vitro digestibility, dried marigold flowers were purchased from a farm in Lampang Province, Thailand.

#### 2.1.2. Proximate Chemical Composition

The proximate compositions of YB and marigolds, including moisture, ash, crude protein, crude lipid, crude fiber, and nitrogen-free extract (NFE), were determined according to the AOAC method [25]. Moisture content was determined by drying samples for 24 h at 105 °C in a hot air oven (WOF155; Wisd Laboratory Instruments, Wertheim, Germany). To determine ash content, samples were burned for 2 h at 600 °C in a muffle furnace (E30-HT; Thai Furnaces Engineering, Lampang, Thailand). Crude protein was determined by using a Kjeldahl analyzer (Kjeltec™ 8100, FossTecator, Höganäs, Sweden). To determine the crude lipid content, a Soxhlet extraction unit (Soxtec™ 8000; Foss, Suzhou, China) was used with petroleum ether as the solvent. Crude fiber, based on acid and alkali digestion, was estimated using a fiber analyzer (F800; Hanon Advanced Technology Group, Jinan, China). The NFE (%) was calculated as [100 − (moisture + ash + crude protein + crude lipid + crude fiber)].

#### 2.1.3. Total Carotenoid Content

The total carotenoid contents of YB and marigolds was determined using the method described by Thongprajukaew et al. [26]. Carotenoids were extracted by mixing 3 mg of freeze-dried sample with 1 mL of acetone. The sample was extracted in darkness at 4 °C for 3 days, with gentle shaking performed daily. The sample was then centrifuged at 5000× *g* for 10 min at room temperature, and 1 mL of the supernatant was aspirated and measured with a spectrophotometer at 474 nm. The total carotenoid content was calculated using the equation *E*_(1%, 1 cm)_ = 1900 [27] and expressed as milligrams per gram of sample.

#### 2.1.4. In Vitro Digestibility

Nile tilapia (5.73 ± 0.05 g body weight, *n* = 4) were purchased from a private farm in Songkhla Province, Thailand. The fish were subsequently euthanized with clove oil, and the intestines were taken out and placed on ice. The intestinal samples were homogenized with cold 0.2 M phosphate buffer, pH 8 (1:3 *w*/*v*), using a tissue homogenizer (D500 Pro; Wiggens GmbH, Wuppertal, Germany). The homogenates were centrifuged at 15,000× *g* for 30 min at 4 °C. The collected supernatants were dialyzed overnight and stored at −20 °C before determination of in vitro digestibility.

The in vitro digestibility reaction was assayed according to the method described by Thongprajukaew et al. [28]. The initial reaction mixes, which included 5 mg of YB or powdered marigold, 10 mL of 50 mM phosphate buffer (pH 8), 50 μL of 0.5% chloramphenicol, and 125 μL of enzyme extract, were incubated for 24 h at 25 °C. The enzymatic reaction was stopped by heating at 100 °C for 10 min. In vitro protein digestibility was determined by comparing the linear range of *DL*-alanine to the increase in released reactive amino groups of cleaved peptides at 420 nm. In vitro carbohydrate digestibility was spectrophotometrically determined at 540 nm by quantifying liberated reducing sugars against a maltose standard curve.

### 2.2. Production of Experimental Diets

To examine the impact of YB inclusion levels in the diet of Nile tilapia, six isonitrogenous, isolipidic, and isoenergetic diets were formulated. The weight percentages of YB used to produce the six experimental diets, including a non-inclusion diet, were 0% (0YB), 2% (2YB), 4% (4YB), 6% (6YB), 8% (8YB), and 10% (10YB). Each dietary formulation contained approximately 40% protein. The dietary content of broken rice was reduced in accordance with the increase in dietary content of YB powder, while other ingredients remained constant. All dry ingredients were finely ground, passed through a 100-mesh sieve, and carefully weighed before being combined in a mixer for 30 min. Lipid sources (soybean oil and fish oil) were added gradually while mixing. To obtain a moist batter, distilled water was added, making up 40% of the weight of the feed. The homogeneous mixture was pressed into pellets with a diameter of 4 mm, that were dried at 60 °C in a hot air oven until the moisture content was reduced to below 10% by weight. The dried pellets were packed in polyethylene bags and stored in a freezer at −20 °C. The chemical composition of each experimental diet was analyzed following the procedure used for YB and marigold samples. Gross energy (kJ/g) values were calculated as [(crude protein × 23.6) + (crude lipid × 39.5) + (NFE × 17.2)]/100.

### 2.3. Fish Feeding Trial

Forty-five-day-old Nile tilapia (*O. niloticus*) were purchased from Songkhla Inland Aquaculture Research and Development Center, Songkhla, Thailand. During their two-week acclimation, the fish were reared in a 1000 L plastic tank (1.46 m diameter × 0.85 m height) and fed a control diet (0YB) twice daily (08:30 and 16:30 h). After the acclimation period, 270 fish with a screened weight of 0.74 ± 0.01 g were equally distributed at random into 18 fiberglass tanks (82 cm width × 55 cm length × 48 cm height) containing 100 L of freshwater. There were 18 experimental units, comprising six dietary groups of fifteen fish each, and three replications. During an eight-week trial, the fish were fed to satiety with an experimental diet twice a day, from 08:30 to 09:00 h and from 16:30 to 17:00 h. The feeding trial was carried out under the natural photoperiod. The tanks were aerated continuously, with daily changes of 80% of the water and daily sediment aspiration. Water temperature and dissolved oxygen measurements were taken using a YSI 550A dissolved oxygen meter (Yellow Spring Instrument, Yellow Spring, OH, USA). Water pH was measured using a pH meter. Ammonia nitrogen in the tank water was assessed using the phenate method according to Boyd and Tucker [29]. The parameters monitored included temperature 26.3 ± 0.1 °C (min–max = 26.3–26.4 °C), dissolved oxygen 6.32 ± 0.03 mg/L (6.25–6.41 mg/L), pH 6.91 ± 0.05 (6.73–7.06), and ammonia nitrogen 1.41 ± 0.05 mg/L (1.27–1.58 mg/L). Mortality and morbidity were monitored every day. After a 30 min meal, uneaten feed—including waste feed—was taken out and dried for 48 h at 60 °C. The amount of feed consumed was determined by computing the weight difference between the given feed and the uneaten feed. These values were utilized to calculate the parameters of feed conversion as follows:

Feeding rate (FR, % body weight/day) = C/[(W_0_ + W_t_)/2]/t × 100 where C = daily feed consumption (g), W_0_ = initial body weight (g), W_t_ = final body weight (g), and t = feeding duration (day),

Feed conversion ratio (FCR) = dry feed consumed (g)/wet weight gain (g), and

Protein efficiency ratio (PER) = wet weight gain (g)/protein intake (g).

### 2.4. Specimen Collection and Biometric Measurements

At the end of the eight-week feeding trial, fish were fasted for 24 h and then anesthetized with clove oil. The skin coloration of fish (*n* = 45 per treatment) was analysed immediately. Then, the final body weight and final total length of fish (*n* = 45 per treatment) were recorded to calculate specific growth rate (SGR), weight gain (WG), and condition factor (CF). Due to the small fish size, blood samples from several fish within the same tank were pooled to obtain one composite sample for hematological analysis (*n* = 3 pooled samples per treatment). The stomach, intestine, and hepatopancreas (*n* = 9 per treatment) were removed, placed on ice and weighed to calculate the stomasomatic index (SSI), intestosomatic index (ISI), and hepatosomatic index (HSI), respectively. Only the gastrointestinal tract was utilized to measure the activities of digestive enzymes, and only the intestine was used in the determination of antioxidant capacity (*n* = 9 per treatment). The fillet color was evaluated from epaxial white muscle taken from below the dorsal fin (*n* = 6 per treatment) from the same fish samples. The same muscle samples were used to determine myosin and actin concentration, and protein synthesis capacity. The whole-body samples of other specimens (*n* = 6 per treatment) were used for chemical composition analysis. All specimens and samples were kept at −20 °C until analysis. The following formulas were used to determine the parameters characterizing survival and growth performances:

Survival (%) = [final fish number/initial fish number] × 100;

SGR (% body weight/day) = [(ln W_t_ − ln W_0_)/(t − t_0_)] × 100 where W_t_ = mean weight (g) at day t, W_0_ = mean weight (g) at day t_0_;

WG (g) = final weight (g) − initial weight (g);

CF = [body weight (g)/total length (cm)^3^] × 100;

SSI (%) = [wet weight of stomach (g)/body weight (g)] × 100;

ISI (%) = [wet weight of intestine (g)/body weight (g)] × 100;

HSI (%) = [wet weight of liver (g)/body weight (g)] × 100.

### 2.5. Color Parameters

The color of skin and epaxial white muscle samples was measured using a MiniScan EZ (Hunter Associates Laboratory, Reston, VA, USA). The black and white standards were used to calibrate the equipment. The color values measured were lightness (L*), redness (a*), chroma (C*), and hue (h*). The L* coordinate is nominally between 0 (darkness) and 100 (brightness), a* represents the degree of redness (+a) to greenness (−a), h* indicates color differences caused by wavelength from red (0°) to blue (270°), and C* is for color purity from vivid (+*C*) to dull (−*C*).

### 2.6. Gut Functionality

#### 2.6.1. Digestive Enzyme Activities

The stomach and intestine samples were homogenized using a tissue homogenizer (D500 Pro; Wiggens GmbH, Wuppertal, Germany) and cold 0.2 M phosphate buffer (pH 8, 1:3 *w*/*v*). After that, the mixture was centrifuged for 30 min at 4 °C at 15,000× *g*. Before the digestive enzyme activity were measured, the collected supernatants were kept at −20 °C. The protein concentration in the crude enzyme extracts was determined following the method described by Lowry et al. [30], using bovine serum albumin as the standard. The concentration of protein (mg/mL) was utilized to measure enzyme specific activity (U/mg protein).

Intestinal extracts were used to evaluate the activities of amylase, cellulase, trypsin, and chymotrypsin whereas pepsin activity was evaluated using stomach extracts. All enzyme activity assays were performed under the assay conditions previously described by Hahor et al. [31]. The activity of α-amylase was assayed using soluble starch as a substrate, according to Areekijseree et al. [32]. Cellulase activity was measured using carboxymethylcellulose as a substrate [33]. The activity of pepsin was determined using hemoglobin as the substrate, following the method described by Worthington [34]. Trypsin and chymotrypsin activities were estimated based on Rungruangsak-Torrissen et al. [35], using *N*-benzoyl-*L*-Arg-*p*-nitroanilide (BAPNA) and *N*-succinyl-Ala-Ala-Pro-Phe-*p*-nitroanilide (SAPNA) as the respective substrates. One unit (U) of amylase, cellulase, trypsin, and chymotrypsin, was defined as the concentration that catalyzed the conversion of 1 μmol of substrate per minute, whereas one U of pepsin was defined by an increase in absorbance of 1.0 at 280 nm. The amylase/trypsin ratio (A/T ratio) was calculated using the specific amylase and trypsin activities from the same sample.

#### 2.6.2. Radical Scavenging Activity

2,2-Diphenyl-1-picrylhydrazyl (DPPH) radical scavenging activity was determined by using intestinal extracts according to the method of Thongprajukaew et al. [36]. Briefly, a stock solution of 0.2 mM DPPH in ethanol was prepared to a concentration of 1.0 ± 0.5 units based on the absorbance measured at 517 nm. The assay was performed with a sample extract–DPPH solution ratio of 0.6 mL:0.6 mL. The mixture was incubated in darkness at room temperature for 30 min, and absorbance was measured at 517 nm. The radical scavenging activity (% inhibition) was calculated from [(A_0_ − A_i_)/A_0_] × 100, where A_0_ is the absorbance of the buffer extraction and A_i_ is the absorbance of the extract sample.

2,2′-Azino-bis (3-ethylbenzthiazoline-6-sulphonic acid) (ABTS) radical scavenging activity was determined following the method described by Re et al. [37] with some modifications. A stock solution was prepared by mixing 7 mM ABTS^+^ with 2.4 mM potassium persulfate (1:1 *v*/*v*). The stock solution was incubated in darkness for 16 h at room temperature to produce a working solution. ABTS^+^ was diluted with distilled water until absorbance at 734 nm was in the range of 1.0 ± 0.5 units. The assay was performed by mixing the sample extract with ABTS^+^ at a ratio of 80 µL:1 mL. The absorbance was measured at 734 nm, and the radical scavenging activity (% inhibition) was calculated from [(A_0_ − A_i_)/A_0_] × 100, where A_0_ is the absorbance of the buffer extraction and A_i_ is the absorbance of the extract sample.

### 2.7. Determination of Fillet Quality

#### 2.7.1. Myosin and Actin Contents

The thermal properties of white muscle samples were investigated using differential scanning calorimetry (DSC7; PerkinElmer, Waltham, MA, USA). Ten milligrams of sample were placed in an aluminum cassette and sealed. An empty cassette was used as a reference pan. The sample was heated from 20 to 120 °C at 10 °C/min, using ice as a cooling source. Myosin and actin contents were characterized based on the onset temperature (T_o_), peak temperature (T_p_), and endset temperature (T_c_) of their thermal transitions. The area under the thermogram peak was defined as the enthalpy change (ΔH), representing the amount of heat required for protein denaturation.

#### 2.7.2. RNA and Protein Concentrations

The concentrations of RNA and protein in white muscle samples were determined based on the method of Rungruangsak-Torrissen [38]. Briefly, approximately 50 mg of frozen white muscle was sonicated with TRIzol^®^ reagent (Invitrogen, Carlsbad, CA, USA) and chloroform was added to separate the organic phase (protein) and aqueous phase (RNA). These phases were precipitated using isopropanol, washed using ethanol, and then oven-dried. The organic phase was dissolved with sodium dodecyl sulfate and measured spectrophotometrically at 280 nm. The aqueous phase was dissolved with sodium acetate, and its absorbance was measured at 260 nm. The extinction coefficients used to calculate the concentrations of protein and RNA were *E*_280_ = 2.1 mg/mL and *E*_260_ = 40 µg/mL, respectively.

### 2.8. Determination of Hematological Parameters

Whole blood was taken from the caudal vessel with 1 mL syringes (needle size 25G; Nipro, Miami, FL, USA) and then transferred to heparinized tubes to assay red blood cells (RBC), white blood cells (WBC), packed cell volume (PCV), and hemoglobin (Hb). A hemacytometer (Bright-Line™, Hausser Scientific, Horsham, PA, USA) was used to count RBC and WBC under a compound microscope. PCV and Hb levels were determined using the cyanohemoglobin technique [39,40]. Lysozyme activity was measured following the method described by Demers and Bayne [41] using a suspension of 0.075% lyophilized *Micrococcus lysodeikticus* (Sigma-Aldrich, St. Louis, MO, USA) in 0.1 M phosphate-citrate buffer (pH 5.8) as the substrate. Briefly, 25 µL of serum was mixed with 175 µL of substrate in a flat-bottomed 96-well plate. The absorbance at 450 nm was measured kinetically every minute for 5 min using a Multiskan™ GO microplate spectrophotometer (Thermo Fisher Scientific, Waltham, MA, USA). Lysozyme activity was defined as a decrease in optical density of 0.001 per minute and expressed as units of enzyme activity (U/mg protein). Plasma protein was measured using bovine serum albumin as the protein standard in accordance with Lowry et al. [30]. Mean cell volume (MCV), mean cell hemoglobin (MCH), and mean cell hemoglobin concentration (MCHC) were calculated as follows:

MCV (fL) = 10 × [Hct (%)/RBC (×10^6^ cells/μL)];

MCH (pg) = 10 × [Hb (g/dL)/RBC (×10^6^ cells/μL)];

MCHC (g/dL) = 100 × [Hb (g/dL)/Hct (%)].

### 2.9. Determination of Whole-Body Composition

The whole-body specimen was minced to homogeneity. The proximate composition of the fish carcass, in terms of moisture, crude protein, crude lipid, and ash, were analyzed as described by the AOAC [25]. All components were determined based on wet weight.

### 2.10. Statistical Analysis

This feeding trial was based on a Completely Randomized Design. The Statistical Package for the Social Sciences (SPSS) Version 17 (SPSS Inc., Chicago, IL, USA) was applied for the statistical analysis. The results were reported in terms of tank means ± standard error (SE). *T*-tests were applied to compare the differences in in vitro digestibility between YB and marigolds. One-Way Analysis of Variance was used to compare the significant differences between means in the feeding trial. Duncan’s multiple range test at the 95% confidence level was used as the post hoc test. Letter-based significance notations presented in the tables were assigned according to the homogeneous subsets generated from Duncan’s multiple range test.

## 3. Results

### 3.1. Nutritional Value of YB and Marigold Flowers and Proximate Composition of Experimental Diets

Marigold flowers contained greater percentages of crude protein, crude lipid, crude fiber, ash, and total carotenoids than YB, while YB contained a greater percentage of NFE. The in vitro digestibility assays revealed that protein digestibility was significantly higher in marigold flowers (*p* = 0.009) but carbohydrate digestibility was lower (*p* < 0.001, Table 1). The proximate chemical compositions of the six experimental diets containing graded levels of YB are presented in Table 2. All diets were formulated to be isonitrogenous, isolipidic, and isoenergetic, with crude protein ranging from 38.4 to 41.3%, crude lipid from 4.67 to 5.23%, and gross energy from 16.7 to 17.5 kJ/g.

### 3.2. Survival, Growth, and Feed Utilization

Dietary supplementation with YB did not affect survival, which was in the range of 97.8 to 100% (*p* = 0.701, Table 3). Final body weight and WG were not different in the 0YB, 2YB, 4YB, 6YB and 8YB treatments but decreased in the 10YB treatment, as did final total length when compared to the control (0YB) (*p* = 0.001). Although WG differed among treatments, SGR was not significantly affected by dietary YB supplementation (*p* = 0.273). Compared with the 0YB treatment, SSI (*p* < 0.001) and ISI (*p* = 0.004) values were higher in the 10YB and 8YB treatments, respectively. HSI values did not differ from the control in any of the treatments, but differed between treatments (*p* = 0.004). However, YB supplementation had no effect on CF (*p* = 0.622) and feed utilization, in terms of FR (*p* = 0.494), FCR (*p* = 0.429) and PER (*p* = 0.346).

### 3.3. Skin and Fillet Coloration

Supplementation with YB did not affect skin and fillet coloration parameters. Lightness, redness, chroma, and hue in skin varied from 42.2 to 46.5 (*p* = 0.498), −3.05 to −3.91 (*p* = 0.999), 3.61 to 4.76 (*p* = 0.862), and 146 to 182 (*p* = 0.780), respectively. These same parameters varied from 44.9 to 52.3 (*p* = 0.157), −2.45 to −3.91 (*p* = 0.690), 4.11 to 4.81 (*p* = 0.800), and 146 to 233 (*p* = 0.144) in fish fillet.

### 3.4. Digestive Enzyme Activities and Gastrointestinal Radical Scavenging Activity

**Figure 1:**
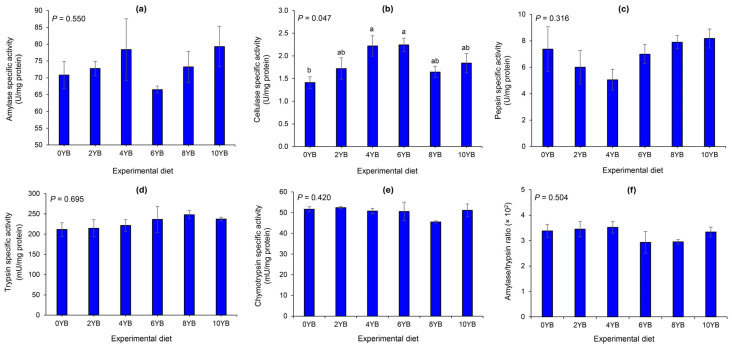
Effects of dietary yellow bells flower (YB) supplementation on digestive enzyme activities in Nile tilapia after 8 weeks. The charts show the specific activities of amylase (**a**), cellulase (**b**), pepsin (**c**), trypsin (**d**), chymotrypsin (**e**), and amylase/trypsin ratio (**f**). Data are expressed as means ± SE from three replicates. Different superscript letters indicate different homogeneous subsets according to Duncan’s multiple range test (*p* < 0.05). The number in the label of each experimental diet indicates the percentage of YB included by weight.

Compared with control, there were no differences in amylase specific activity across the five YB dietary treatments (*p* = 0.550, Figure 1a). Cellulase specific activity was higher in the 4YB and 6YB groups compared with the control (*p* = 0.047, Figure 1b). Other dietary levels provided overlapping cellulase values. Specific activities of pepsin (*p* = 0.316, Figure 1c), trypsin (*p* = 0.695, Figure 1d), and chymotrypsin (*p* = 0.420, Figure 1e), as well as the amylase/trypsin ratio (*p* = 0.504, Figure 1f), did not differ between treatments.

**Figure 2:**
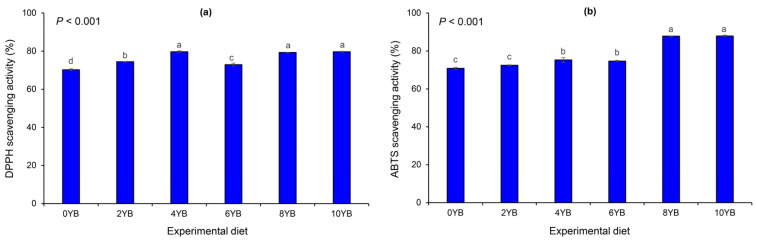
Effects of dietary yellow bells flower (YB) supplementation on scavenging activities in Nile tilapia after 8 weeks. The charts show the radical scavenging activity in intestinal crude extracts of the fish: DPPH (**a**) and ABTS (**b**). Data are expressed as means ± SE from three replicates. Different superscript letters indicate different homogeneous subsets according to Duncan’s multiple range test (*p* < 0.05). The number in the label of each experimental diet indicates the percentage of YB included by weight. DPPH = 2,2-diphenylpicrylhydrazyl, ABTS = 2,2-azino-bis (3-ethylbenzothiazoline-6-sulfonic acid).

DPPH scavenging activity (*p* < 0.001, Figure 2a) was significantly higher in the YB treatments compared with the control. The highest levels were observed in the 4YB, 8YB, and 10YB groups followed by the 6YB and 2YB groups. ABTS scavenging activity tended to increase with supplementation levels (*p* < 0.001, Figure 2b).

### 3.5. Fillet Quality

Myosin contents were highest in the 4YB and 6YB treatments and significantly lower than control in the 8YB treatment (*p* = 0.001, Table 4). Actin contents (*p* = 0.315) and the actin/myosin ratio (*p* = 0.236) were not different across treatments, while the sum of myosin and actin was highest in the 4YB treatment but low in the 2YB, 8YB and 10YB treatments (*p* < 0.001). No differences in RNA concentration (*p* = 0.307), protein concentration (*p* = 0.761), and RNA/protein ratio (*p* = 0.292) were observed in the fillet of fish across the six dietary groups (Table 4).

### 3.6. Hematological Parameters

WBC counts were lower in all treatments compared with the control (*p* = 0.030, Table 5). PCV values were higher than control in all treatments (*p* = 0.011) except the 10YB treatment which did not differ from the control. Hb values showed similar changes, with values not differing from control in the 2YB and 10YB treatments. Lysozyme activity was significantly higher in fish fed the 4YB diet compared to the control and 10YB (*p* = 0.011), but not significantly different from those fed the 2YB, 6YB, or 8YB diet (*p* > 0.05). The other parameters, RBC (*p* = 0.057), MCV (*p* = 0.862), MCH (*p* = 0.865), MCHC (*p* = 0.649), thrombocytes (*p* = 0.393), and plasma protein (*p* = 0.217), were not different among the six groups.

### 3.7. Whole-Body Composition

Moisture (*p* = 0.554) and crude lipid (*p* = 0.441) levels remained consistent across all dietary groups throughout the feeding trial (Table 6). In contrast, whole-body ash content increased significantly in the 4YB, 8YB, and 10YB treatments compared to the control (*p* = 0.024). Fish that received higher inclusion levels exhibited elevated ash contents, suggesting an enhanced mineral deposition associated with YB supplementation. Whole-body crude protein differed significantly among treatments (*p* = 0.046). Fish in the 10YB treatment exhibited the highest protein level, followed by fish in the 8YB and 4YB treatments.

## 4. Discussion

YB had lower levels of nutritional components than marigolds but the NFE and carbohydrate digestibility of YB were 1.59 and 9.68 times higher, respectively. The nutritional components were of secondary importance as this study focused on the biological activity of YB, which may be beneficial for aquaculture. Based on our study, dietary supplementation with 4% YB appeared to provide the most favorable overall responses in Nile tilapia across the assessed parameters. However, there is limited specific data on the precise levels of YB in animal feed, but studies indicate that the plant itself, including its leaves and flowers, can serve as a nutritious animal fodder [42]. The proposed YB supplementation level in the current study is similar to the appropriate inclusion of marigold flowers in aquatic animal feed which varies by species and purpose. The appropriate supplementation ranges from 1.5% to 4% marigold meal to achieve the beneficial effects of desirable pigmentation, growth, and immune responses [43,44].

The reduced growth coupled with elevated SSI/ISI in 8YB–10YB groups indicates energy reallocation toward digestive organ development at the expense of somatic growth. This pattern may also reflect a hormetic response, in which moderate YB supplementation exerts beneficial physiological effects, whereas excessive supplementation may impose metabolic costs associated with higher fiber content or excess phytochemical intake. Although WG decreased in the 8YB and 10YB treatments, SGR was not significantly affected by dietary YB supplementation. This discrepancy may be related to the logarithmic nature of SGR calculation, which can be less sensitive to relatively small differences in final body weight among treatments. It has been proposed that fish may actively allocate energy, diverting resources to expand or maintain digestive organs to deal with complex digestion demands, rather than directing energy exclusively toward somatic growth [45]. On the other hand, low to moderate supplementation with YB (2YB to 4YB) maintained good growth and balanced SSI or ISI values. The HSI values observed at moderate levels of YB supplementation (4YB to 6YB) may reflect slightly increased hepatic energy reserves, whereas lower or higher supplementation levels showed less pronounced responses. Liver/lipid/glycogen tests or histopathology would be required to produce a definitive liver health profile.

The unchanged FR, FCR, and PER values indicated that fish in the control and all treatments consumed and utilized their diets in the same way. However, the fish that received YB had better digestive system health and functions. The improved radical scavenging activity observed in all fish receiving YB may be associated with the presence of alkaloids, flavonoids, phenols, saponins, and quinones that are responsible for the antioxidant qualities of YB flowers [46]. Moreover, this plant and other plants in the genus Tecoma contain a variety of bioactive compounds that can treat diseases related to the digestive system [4,5,6,7,8].

YB did not affect starch-digesting enzymes, as evidenced by the unchanged specific amylase activity. However, cellulase specific activity varied depending on the supplementation level. Although YB does not directly stimulate cellulase, the flavonoids and phenolic compounds in the flower may modulate gut microflora and reduce pathogenicity [4,12], indirectly enabling cellulase-producing microorganisms to work more efficiently. Therefore, the appropriate supplementation (4YB to 6YB) may increase the effective microbial population in the gut, resulting in higher cellulase activity. Improved cellulase activity together with enhanced gastrointestinal radical-scavenging capacity may indicate improved gut functional status in fish receiving moderate levels of YB supplementation. These responses may partly explain the favorable physiological responses and fillet quality parameters observed in the present study. However, excessive supplementation (8YB to 10YB) may have the opposite effect, as some phenolic compounds could disrupt the microflora or cause nutritional resistance, resulting in a slight decrease in cellulase activity. Such responses suggest that dietary YB may influence intestinal microbial balance and antioxidant-related physiological processes in Nile tilapia.

Since the active ingredients in YB may not directly affect the secretion or function of pepsin, trypsin, and chymotrypsin, or the amounts may not be sufficient to do so, adding YB to the diet of tilapia may not significantly alter the activities of these enzymes. The amylase/trypsin ratio is a measure of the ability of the gastrointestinal tract to process different meal components; a high ratio often denotes more carbohydrate digestibility, whereas a low ratio denotes better protein digestibility [28]. Thus, the absence of significant variation in this parameter across the treatments suggests that the balance of protein and carbohydrate use was unaffected by YB.

Fish blood parameters, both hematological and biochemical, serve as critical indicators of health, stress, and environmental quality in aquaculture and research and are influenced by species, sex, nutrition, and seasonal changes [47,48,49]. In this study, only three of the nine parameters were found to be affected by dietary YB supplementation. The addition of some nutritional feed additives reduces stress and inflammation, and hence the quantities of WBCs produced by fish, which is considered beneficial [50]. Therefore, the reduction in WBCs in observed in fish receiving YB can be considered a positive result of this trial. The PCV is a biomarker that reflects the proportion of RBCs in the blood, which is related to the ability of the blood to transport oxygen to tissues. Changes in PCV often indicate blood conditions such as anemia, hemolysis, or response to stress/environmental factors [51,52,53]. Hb is the main protein in RBCs that binds and transports oxygen from the gills to tissues throughout the body and carries carbon dioxide back to the gills for removal. Given the unaltered RBC density but elevated Hb and PCV levels in comparison to the control, the 4YB to 8YB treatments facilitated oxygen delivery and blood circulation. Lysozyme in fish serum serves as a critical innate immune enzyme that hydrolyzes peptidoglycan in bacterial cell walls, providing frontline defense against microbial infections [54]. The present study suggests that total phenolic compounds from YB flowers enhance serum lysozyme activity primarily through antioxidant and immunomodulatory mechanisms, including macrophage and phagocyte activation, upregulated lysozyme gene expression, elevated complement activity and phagocytosis, and suppressed pro-inflammatory cytokines [55,56]. Because the experimental fish were small, blood samples from multiple fish within the same tank were pooled to obtain sufficient sample volume for hematological analyses. Therefore, the observed responses should be interpreted primarily at the tank level, and subtle individual physiological variations among fish may not have been fully detected.

Since the carotenoid content of YB was lower than that of marigolds, no changes in the color of tilapia, either in the skin or in the fillet were anticipated or observed. However, some significant changes occurred in fillet quality. The findings indicate that dietary supplementation with the right amount of YB could raise fish myosin content and total amounts of myosin and actin when compared with the control. Therefore, YB may help maintain muscle structural proteins and preserve muscle fiber stability. Muscle cells can preserve an environment that is conducive to the synthesis of myofibrillar proteins such as actin and myosin, by minimizing damage from free radicals, and the flowers of YB are abundant in phytochemicals with anti-inflammatory and antioxidant qualities [4,5,6,7,9,10,11]. Muscle firmness and meat quality, including flexibility, hydration retention, and resistance to deterioration, are enhanced by higher protein content [57]. Given that consumers typically seek firm, fresh flesh, this is significant for the value of farmed fish. The pro-oxidant effect of the phytochemicals when taken in excess or the disruption of normal cellular metabolism [58] may have been the cause of the tendency of these parameters to decrease in the 8YB and 10YB treatments, which prevented further increases in structural protein accumulation.

Myosin content declined at higher inclusion levels of YB (8YB–10YB); however, this pattern was not accompanied by changes in RNA concentration, protein concentration, or the RNA/protein ratio. This apparent discrepancy suggests that the reduction in myosin content may be attributed to protein turnover dynamics, structural protein remodeling, or selective protein degradation rather than an overall decline in protein synthesis capacity. Recent studies have indicated that myofibrillar proteins, including myosin, can respond to dietary bioactive compounds or metabolic stress through changes in muscle protein turnover and quality traits, even when global indices of protein synthesis remain unchanged [59,60]. Because RNA concentration and the RNA/protein ratio primarily reflect ribosomal abundance and translational potential, their stability in this study indicates that the muscle maintained its general protein synthetic machinery despite the reduction in specific contractile proteins. Similar changes in muscle quality and biochemical composition have been documented in fish fed functional or phytochemical-enriched diets. These responses suggest that dietary bioactive compounds may modulate muscle protein turnover and flesh quality traits, although direct evidence for altered translational activity or specific myofibrillar protein remodeling remains limited [59,61]. Flavonoids and other antioxidant compounds in YB may therefore influence muscle quality through post-transcriptional regulation, altered protein turnover rates, or stabilization of myofibrillar proteins rather than through changes in overall transcriptional activity. Therefore, the observed response in this study may reflect indirect effects of YB bioactive compounds on muscle metabolic processes.

The whole-body composition of Nile tilapia was only partly affected by dietary YB supplementation. Moisture and crude lipid did not differ among treatments, indicating that YB had no substantial influence on body water balance or lipid deposition. Similar findings have been reported in Nile tilapia fed phytogenic additives, where growth or health responses improved without marked changes in whole-body lipid content [62,63]. In contrast, crude protein content was significantly higher at the higher YB supplementation levels, with the greatest values observed in the 10YB treatment. These findings suggest improved protein retention—potentially linked to the antioxidant and phytochemical components of YB that may enhance nutrient utilization, reduce oxidative stress, and support lean tissue deposition in fish fed phytogenic additives [64,65]. Ash content also varied significantly, which may reflect changes in mineral deposition and nutrient utilization associated with dietary phytogenic additives rich in flavonoids and other bioactive compounds [63]. Overall, these results suggest that while YB does not markedly influence lipid or moisture composition, higher inclusion levels can promote protein and mineral accumulation, supporting its potential as a functional phytogenic additive in tilapia nutrition.

## 5. Conclusions

This study demonstrated that the dried flower of the yellow bells plant (*Tecoma stans*; YB) can be used as a functional feed additive for Nile tilapia (*Oreochromis niloticus*). Although YB contained less crude protein and lipid than marigold, its higher carbohydrate digestibility allowed its inclusion at up to 8% of the diet by weight without compromising survival, growth, feed conversion, or skin and fillet coloration. Dietary YB markedly enhanced gut functionality, as evidenced by increased intestinal cellulase activity and strong radical-scavenging activities, while amylase and protease activities and the amylase/trypsin ratio remained unchanged. At moderate inclusion levels, hematological markers indicated improved physiological status, with reduced white blood cell counts and elevated packed cell volume and hemoglobin levels, without adverse changes in other blood indices. Fillet quality was also improved, since 4% YB increased myosin and total myofibrillar protein content without altering RNA, total protein, or the RNA/protein ratio. At higher levels of YB supplementation, whole-body moisture and crude lipid percentages were unaffected, whereas crude protein and ash percentages were higher. Since the trial was short and performed under controlled conditions, responses in other life stages or farming systems remain uncertain. In addition, the observed improvements in gut functionality and physiological parameters should be interpreted with caution, as a minor contribution from dietary protein variation among diets cannot be completely excluded. However, overall, a dietary inclusion level of around 4% appeared to provide the most favorable responses in gut functionality, fillet quality, blood parameters, and whole-body composition in Nile tilapia.

## Figures and Tables

**Table 1:** The proximate chemical compositions (dry weight basis) and in vitro digestibility of dried yellow bells and marigold flowers are compared. The values are expressed as means from triplicate samples.

Component	Yellow Bells	Marigold
*Chemical composition*		
Crude protein (%)	13.2	19.9
Crude lipid (%)	4.77	8.41
Crude fiber (%)	9.87	11.2
Ash (%)	4.28	6.41
Nitrogen-free extract (%)	65.6	41.2
Carotenoid (mg/g)	1.18	1.40
In vitro *digestibility*		
Protein (mmol *DL*-alanine equivalent/g)	24.7 ± 0.9 ^b^	29.0 ± 0.4 ^a^
Carbohydrate (μmol maltose/g)	438 ± 31 ^a^	45.2 ± 2.3 ^b^

Different superscripts in the same row indicate significant differences according to Student’s *t*-test (*p* < 0.05).

**Table 2:** Formulations and proximate chemical compositions of experimental feeds containing different levels of dried yellow bells flower (YB).

Ingredient and Chemical Component	Experimental Diet					
	0YB	2YB	4YB	6YB	8YB	10YB
*Ingredient (% of feed)*						
Fish meal	28.0	28.0	28.0	28.0	28.0	28.0
Soybean meal	28.0	28.0	28.0	28.0	28.0	28.0
Wheat flour	25.4	25.4	25.4	25.4	25.4	25.4
Broken rice	10.0	8.00	6.00	4.00	2.00	0.00
Soybean oil	1.75	1.75	1.75	1.75	1.75	1.75
Fish oil	1.75	1.75	1.75	1.75	1.75	1.75
Di-calcium phosphate	1.00	1.00	1.00	1.00	1.00	1.00
Choline chloride	0.10	0.10	0.10	0.10	0.10	0.10
Vitamin-mineral premix ^1^	2.00	2.00	2.00	2.00	2.00	2.00
Carboxymethylcellulose	2.00	2.00	2.00	2.00	2.00	2.00
Dried yellow bells flower	0.00	2.00	4.00	6.00	8.00	10.0
*Proximate composition (* *as-fed basis* *)*						
Moisture (%)	11.2	11.6	11.1	8.96	8.70	10.1
Crude protein (%)	38.4	40.9	40.6	41.1	41.3	40.7
Crude lipid (%)	4.67	4.87	4.89	4.97	5.23	5.22
Crude fiber (%)	1.32	1.56	1.75	1.74	1.79	2.10
Ash (%)	10.5	9.85	9.88	10.1	10.1	10.1
Nitrogen-free extract (%)	33.9	31.3	31.8	33.1	32.9	31.8
Gross energy (kJ/g)	16.7	17.0	17.0	17.4	17.5	17.1

^1^ Vitamin-mineral premix provides 4,000,000 IU vitamin A, 450,000 IU vitamin D_3_, 6500 mg vitamin E, 1000 mg vitamin K_3_, 3900 mg vitamin B_1_, 2400 mg vitamin B_2_, 2250 mg vitamin B_6_, 5 mg vitamin B_12_, 15,000 mg vitamin C, 120 mg biotin, 5520 mg niacin, 100,000 mg Ca, 80,000 mg P, 2500 mg Cu, 1200 mg Fe, 1200 mg Mn, 1540 mg Zn, 260 mg K, 740 mg I, 2160 mg Mg, 10 mg Se, and 240 mg Co.

**Table 3:** Survival, growth performance, and feed utilization parameters of Nile tilapia that received experimental diets containing different levels of dried yellow bells flower (YB) for eight weeks.

Parameter	Experimental Diet						*p*-Value
	0YB	2YB	4YB	6YB	8YB	10YB	
Survival (%)	97.8 ± 2.2	100 ± 0	97.8 ± 2.2	100 ± 0	97.8 ± 2.2	100 ± 0	0.701
IBW (g)	0.72 ± 0.01	0.73 ± 0.01	0.74 ± 0.01	0.74 ± 0.02	0.75 ± 0.02	0.74 ± 0.01	0.099
FBW (g)	51.5 ± 0.8 ^ab^	56.0 ± 1.4 ^a^	53.2 ± 1.2 ^a^	52.5 ± 1.8 ^ab^	46.8 ± 3.0 ^bc^	44.1 ± 2.5 ^c^	0.008
FTL (cm)	13.6 ± 0.1 ^ab^	13.9 ± 0.2 ^a^	13.8 ± 0.1 ^ab^	13.5 ± 0.2 ^bc^	12.9 ± 0.1 ^d^	13.1 ± 0.1 ^cd^	0.001
SGR (% BW/day)	2.33 ± 0.05	2.37 ± 0.06	2.28 ± 0.06	2.27 ± 0.06	2.26 ± 0.06	2.18 ± 0.03	0.273
WG (g)	50.2 ± 0.3 ^ab^	54.6 ± 1.6 ^a^	51.3 ± 1.7 ^ab^	50.5 ± 1.4 ^ab^	45.5 ± 3.2 ^bc^	42.2 ± 1.9 ^c^	0.008
CF	1.96 ± 0.03	1.98 ± 0.03	1.94 ± 0.04	2.04 ± 0.02	1.97 ± 0.04	1.92 ± 0.09	0.622
SSI (%)	0.73 ± 0.02 ^bc^	0.69 ± 0.03 ^c^	0.90 ± 0.05 ^b^	0.90 ± 0.03 ^b^	0.82 ± 0.07 ^bc^	1.14 ± 0.10 ^a^	<0.001
ISI (%)	3.44 ± 0.15 ^b^	3.65 ± 0.19 ^b^	3.55 ± 0.19 ^b^	3.84 ± 0.27 ^b^	4.76 ± 0.35 ^a^	3.69 ± 0.16 ^b^	0.004
HSI (%)	0.35 ± 0.04 ^abc^	0.23 ± 0.04 ^c^	0.46 ± 0.05 ^a^	0.45 ± 0.02 ^ab^	0.34 ± 0.03 ^bc^	0.33 ± 0.03 ^c^	0.004
FR (% BW/day)	3.90 ± 0.06	3.85 ± 0.04	3.80 ± 0.14	3.70 ± 0.06	3.84 ± 0.13	4.00 ± 0.14	0.494
FCR	1.12 ± 0.02	1.08 ± 0.03	1.09 ± 0.04	1.06 ± 0.02	1.11 ± 0.04	1.16 ± 0.04	0.429
PER	2.32 ± 0.04	2.26 ± 0.07	2.26 ± 0.08	2.29 ± 0.03	2.19 ± 0.07	2.12 ± 0.07	0.346

IBW, initial body weight; FBW, final body weight; FTL, final total length; SGR, specific growth rate; WG, weight gain; CF, condition factor; SSI, stomasomatic index; ISI, intestosomatic index; HSI, hepatosomatic index; FR, feeding rate; FCR, feed conversion ratio; PER, protein efficiency ratio. Data are expressed as means ± SE. Different superscript letters indicate different homogeneous subsets according to Duncan’s multiple range test (*p* < 0.05).

**Table 4:** The fillet quality of Nile tilapia that received experimental diets containing different levels of dried yellow bells flower (YB) for eight weeks.

Parameter	Experimental Diet						*p*-Value
	0YB	2YB	4YB	6YB	8YB	10YB	
*Myosin and actin contents*							
ΔH Myosin (J/g)	1.78 ± 0.03 ^b^	1.72 ± 0.06 ^b^	2.03 ± 0.08 ^a^	1.94 ± 0.02 ^a^	1.47 ± 0.02 ^c^	1.63 ± 0.05 ^bc^	0.001
ΔH Actin (J/g)	0.37 ± 0.01	0.33 ± 0.06	0.42 ± 0.02	0.33 ± 0.01	0.36 ± 0.01	0.35 ± 0.02	0.315
ƩΔH (J/g)	2.15 ± 0.04 ^b^	1.95 ± 0.04 ^c^	2.43 ± 0.05 ^a^	2.26 ± 0.02 ^b^	1.82 ± 0.01 ^d^	1.99 ± 0.02^c^	<0.001
Actin/myosin ratio	0.21 ± 0.01	0.16 ± 0.01	0.20 ± 0.01	0.16 ± 0.01	0.24 ± 0.01	0.20 ± 0.02	0.236
*Protein synthesis capacity*							
RNA (μg/g)	1648 ± 25	1729 ± 107	1902 ± 96	1728 ± 95	1648 ± 94	1669 ± 71	0.307
Protein (mg/g)	165 ± 29	182 ± 14	192 ± 17	201 ± 31	203 ± 13	200 ± 7	0.761
RNA/protein ratio (μg/mg)	11.6 ± 1.9	9.61 ± 0.57	10.4 ± 1.3	10.4 ± 0.7	8.37 ± 0.94	8.40 ± 0.47	0.292

Data are expressed as means ± SE. Different superscript letters indicate different homogeneous subsets according to Duncan’s multiple range test (*p* < 0.05).

**Table 5:** Hematological parameters of Nile tilapia that received experimental diets containing different levels of dried yellow bells flower (YB) for eight weeks.

Parameter	Experimental Diet						*p*-Value
	0YB	2YB	4YB	6YB	8YB	10YB	
WBC (×10^3^ cells/μL)	2.83 ± 0.42 ^a^	1.67 ± 0.42 ^b^	1.75 ± 0.29 ^b^	1.50 ± 0.25 ^b^	1.25 ± 0.14 ^b^	1.42 ± 0.08 ^b^	0.030
RBC (×10^6^ cells/μL)	1.72 ± 0.11	2.05 ± 0.04	2.07 ± 0.07	2.12 ± 0.11	1.84 ± 0.07	1.90 ± 0.03	0.057
PCV (%)	31.3 ± 1.5 ^c^	35.5 ± 1.0 ^ab^	37.0 ± 1.0 ^a^	36.7 ± 0.3 ^ab^	37.3 ± 0.3 ^a^	33.5 ± 0.5 ^bc^	0.011
Hb (g/dL)	10.4 ± 0.5 ^b^	11.3 ± 0.5 ^ab^	12.3 ± 0.3 ^a^	12.2 ± 0.1 ^a^	12.4 ± 0.1 ^a^	11.7 ± 0.2 ^ab^	0.030
MCV (fL)	173 ± 17	174 ± 13	173 ± 11	185 ± 13	189 ± 14	190 ± 14	0.862
MCH (pg)	57.6 ± 5.5	58.1 ± 4.4	57.7 ± 3.6	57.6 ± 2.1	61.6 ± 4.2	63.0 ± 4.7	0.865
MCHC (g/dL)	33.3 ± 0.1	33.3 ± 0.1	33.3 ± 0.1	33.3 ± 0.1	33.3 ± 0.1	33.3 ± 0.1	0.649
Thrombocytes (×10^3^ cells/μL)	227 ± 44	172 ± 29	237 ± 26	175 ± 9	180 ± 19	185 ± 17	0.393
Plasma protein (g/dL)	2.53 ± 0.07	2.80 ± 0.20	2.73 ± 0.07	2.60 ± 0.01	3.00 ± 0.20	2.80 ± 0.20	0.217
Lysozyme activity (U/mg protein)	22.4 ± 0.9 ^bc^	29.6 ± 1.0 ^abc^	35.0 ± 3.3 ^a^	28.1 ± 1.2 ^abc^	30.1 ± 3.1 ^ab^	20.9 ± 1.4 ^c^	0.011

WBC, white blood cells; RBC, red blood cells; PCV, packed cell volume; Hb, hemoglobin; MCV, mean corpuscular volume; MCH, mean corpuscular hemoglobin; MCHC, mean corpuscular hemoglobin concentration. Data are expressed as means ± SE. Different superscript letters indicate different homogeneous subsets according to Duncan’s multiple range test (*p* < 0.05).

**Table 6:** Whole-body composition (% of fresh weight) of Nile tilapia that received experimental diets containing different levels of dried yellow bells flower (YB) for eight weeks.

Parameter	Experimental Diet						*p*-Value
	0YB	2YB	4YB	6YB	8YB	10YB	
Moisture	73.6 ± 0.3	73.5 ± 0.4	72.7 ± 0.3	73.0 ± 0.2	72.7 ± 0.5	72.7 ± 0.8	0.554
Crude protein	19.3 ± 0.4 ^c^	19.4 ± 0.2 ^c^	20.1 ± 0.2 ^ab^	19.6 ± 0.3 ^bc^	21.0 ± 0.4 ^ab^	21.2 ± 0.9 ^a^	0.046
Crude lipid	6.18 ± 0.21	5.94 ± 0.24	6.08 ± 0.20	5.92 ± 0.28	5.72 ± 0.26	6.46 ± 0.02	0.441
Ash	3.71 ± 0.20 ^c^	4.08 ± 0.38 ^bc^	4.63 ± 0.26 ^ab^	4.08 ± 0.26 ^bc^	4.91 ± 0.18 ^a^	4.85 ± 0.11 ^ab^	0.024

Data are expressed as means ± SE. Different superscript letters indicate different homogeneous subsets according to Duncan’s multiple range test (*p* < 0.05).

## Data Availability

The data presented in this study are available on request from the corresponding author.
